# Gallnuts: A Potential Treasure in Anticancer Drug Discovery

**DOI:** 10.1155/2018/4930371

**Published:** 2018-03-29

**Authors:** Jiayu Gao, Xiao Yang, Weiping Yin, Ming Li

**Affiliations:** ^1^School of Chemical and Pharmaceutical Engineering, Henan University of Scientific and Technology, Henan, China; ^2^School of Clinical Medicine, Henan University of Scientific and Technology, Henan, China; ^3^Luoyang Traditional Chinese Medicine Association, Luoyang, Henan, China

## Abstract

*Introduction. *In the discovery of more potent and selective anticancer drugs, the research continually expands and explores new bioactive metabolites coming from different natural sources. Gallnuts are a group of very special natural products formed through parasitic interaction between plants and insects. Though it has been traditionally used as a source of drugs for the treatment of cancerous diseases in traditional and folk medicinal systems through centuries, the anticancer properties of gallnuts are barely systematically reviewed.* Objective. *To evidence the traditional uses and phytochemicals and pharmacological mechanisms in anticancer aspects of gallnuts, a literature review was performed.* Materials and Methods. *The systematic review approach consisted of searching web-based scientific databases including PubMed, Web of Science, and Science Direct. The keywords for searching include gallnut, Galla Chinensis,* Rhus chinensis*,* Rhus potaninii*,* Rhus punjabensis*, nutgall, gall oak,* Quercus infectoria*,* Quercus lusitanica,* and galla turcica. Two reviewers extracted papers independently to remove the papers unrelated to the anticancer properties of gallnuts. Patents, abstracts, case reports, and abstracts in symposium and congress were excluded.* Results and Conclusion. *As a result, 14 articles were eligible to be evaluated. It is primarily evident that gallnuts contain a number of bioactive metabolites, which account for anticancer activities. The phytochemical and pharmacological studies reviewed strongly underpin a fundamental understanding of anticancer properties for gallnuts (Galla Chinensis and Galla Turcica) and support their ongoing clinical uses in China. The further bioactive compounds screening and evaluation, pharmacological investigation, and clinical trials are expected to progress gallnut-based development to finally transform the wild medicinal gallnuts to the valuable authorized anticancer drugs.

## 1. Introduction

Cancer is a generic term for a vast diversity of diseases that can affect any organ system throughout the body. Though great efforts have been invested over recent decades, the leading morbidity and mortality rates of human cancer have not dramatically changed [1]. The development of more effective anticancer agents thus remains an ongoing challenge. Natural products are the valuable treasury offering approximately 75% of drugs currently used for cancer treatment [2]. With the aim of discovering more potent, more selective, and less toxic compounds than today's drugs, the research of anticancer agents continually expands and explores new natural products coming from different sources, among which the insect gallnuts could be quoted as an almost wild resource rarely systematically studied.

Gallnuts are a group of very special natural products characterized as the plant-insect symbiont. They are formed as the pathological excrescences on the young branches or twigs of plants as a result of the insect attack and deposition of the eggs [3]. Historically, gallnuts have been used by both Western and Eastern cultures as a traditional medicine for various body disorders, as an astringent in painful hemorrhoids, an antiphlogistic for inflammatory conditions, a treatment for diarrhea and dysentery, and a remedy for toothache and dental caries [4, 5]. Galla Chinensis and Galla Turcica are the two most significant gallnuts as a source of medicine widely used in different countries [5].

**Figure 1:**
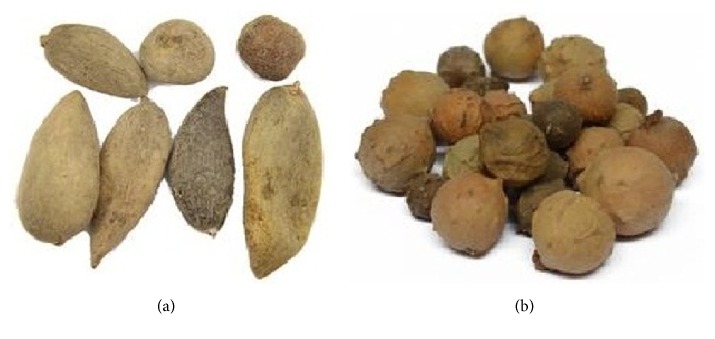
Galla Chinensis (a) and Galla Turcica (b).

Galla Chinensis is formed when the Chinese sumac aphid Baker (mainly* Melaphis chinensis* Bell) parasitizes the leaves or petioles of the plants of family Anacardiaceae (mainly* Rhus chinensis* Mill,* Rhus potaninii* Maxim, and* Rhus punjabensis* var. sinica (Diels) Rehd. Et Wils) [6]. Galla Chinensis is native to and mainly distributed in areas of southern provinces of China, Sumatra, and Malaysia, but it is also produced in small amounts in India, Japan, and Korea [7]. As shown in Figure 1, these gallnuts are typically 4-5 × 1.5 cm in diameter, horned, and reddish-brown in colour, covered with velvety down. The galls are usually harvested between September and October. The medicine Galla Chinensis is the dry and clean gallnuts after removal of the larvae [5, 6].

Galla Turcica is formed on the young branches or twigs of* Quercus infectoria* Olivier parasitized by the gall wasps,* Cynips gallae-tinctoriae* Olivier [8].* Quercus infectoria* Olivier is a small tree or shrub, native to and widely distributed in the Mediterranean coast countries, mainly Greece and Turkey, and also Iran and Syria [5, 8]. Galla Turcica is typically 1–2.5 cm in diameter, being almost spherical in shape, with the tuberculated surface on the upper part. Its colour is gray, white-brown, olive green, or dark bluish-green (Figure 1). The galls of* Quercus infectoria* Olivier are usually harvested between August and September [5].

## 2. Records in TCM Encyclopedia

As the important traditional medicine dates back centuries, the medicinal uses of gallnuts (Galla Chinensis and Galla Turcica) have been widely recorded in more than sixteen classical Traditional Chinese Medicine (TCM) pharmacopeias compiled in different dynasties of China (Table 1). Though those books were written in numerous obscure TCM terminologies, many of recorded uses of gallnuts have been proved for treating clinical features associated with cancer. According to the theories of TCM, cancer is caused by the invasion of exogenous pathogenic factors (accumulated toxins and heat) and the imbalance of endogenous physical conditions (air and blood stasis) [36]. The main therapeutic effects of gallnuts recorded in TCM pharmacopeias are to clear the heat and toxins and rebalance the pathological conditions of human body.

An important medical compendium, *⟪*Tang Materia Medica*⟫* in 659 AD, is the earliest official medical monograph compiled by the state authority in the world [37]. To the best of our knowledge, it is also the first book to record gallnuts medicine (Galla Turcica), as well as their activities in treating diarrhea through eliminating the toxins and rebuilding the normal gastrointestinal environment [9]. The medical uses of the other gallnut, Galla Chinensis, were also firstly recorded to treat the intestinal dysfunction and diarrhea in *⟪*Chinese Materia Medica Gleanings*⟫* at 741 AD [10]. The microenvironment rebalance of digestive system could thus be considered as one of the major therapeutic effects of gallnuts, which was claimed to be a significant progress for cancer treatment in the TCM theories. This therapeutic effect was further recorded by *⟪*Oversea Materia Medica*⟫*, *⟪*Compendium of materia medica*⟫*, *⟪*Fresh Chinese Materia Medica*⟫*, and *⟪*Truth Chinese Materia Medica*⟫* in the following several centuries [13, 19–21].

The other therapeutic effect of gallnuts related to cancer treatment is the pathogen (toxins and heat in TCM theories) scavenging. According to *⟪*Compendium of materia medica*⟫*, one of the most authoritative encyclopedias of TCM, Galla Chinensis could be used in the remedy of phlegm, cough, emesia, analgesic, ulcer, and chancre through clearing the exogenous pathogenic heat and toxins [19]. *⟪*Oversea Materia Medica*⟫*, *⟪*Ri Hua Zi Materia Medica*⟫*, *⟪*Amplification on Materia Medica Addendum*⟫*, and *⟪*Truth Chinese Materia Medica*⟫* also recorded the antipathogenic activity of gallnuts (Galla Chinensis and Galla Turcica) in different periods [13, 14, 17, 21]. Therefore, the accumulated toxins and heat, which were believed as the major carcinogenic factors in TCM theories, could be effectively eliminated under gallnuts treatment.

Taken together, though there's no straight conclusion, the abstracted information from the ancient TCM encyclopedia has highlighted the potentials of gallnuts as the effective anticancer candidates, thus providing an valuable natural resource for further scientific study. A systematic review was performed with the aims of collecting and analyzing current knowledge of anticancer aspects for gallnuts, which may underpin the fundamental understanding and inspire the gallnuts-sourced drug development.

## 3. Articles Selection

**Figure 2:**
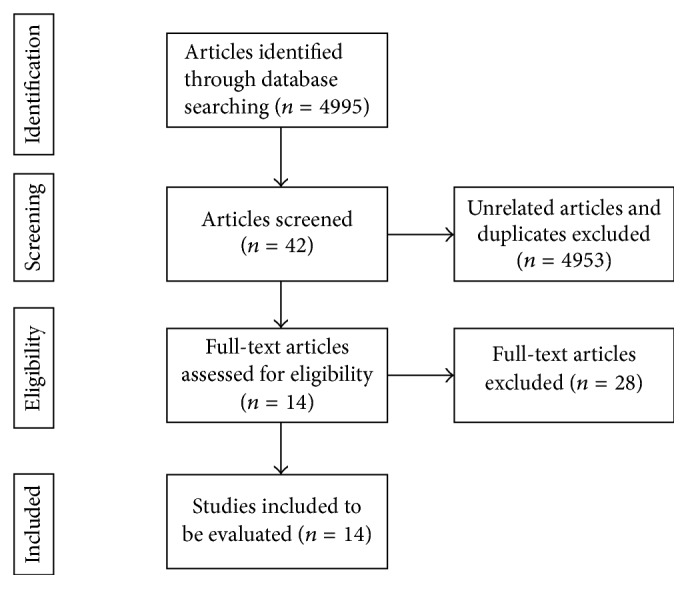
Trend of screening and choosing articles.

The authors searched numbers of electronic databases, including PubMed, Web of Science, and Science Direct up to the date on 31 August 2017. The keywords for searching include gallnut, Galla Chinensis,* Rhus chinensis*,* Rhus potaninii*,* Rhus punjabensis*, nutgall, gall oak,* Quercus infectoria*,* Quercus lusitanica,* and galla turcica. These keywords were searched individually and in combination with cancer and tumour. Searching was limited to articles in either English or Chinese. Two reviewers extracted papers independently. The duplication articles were firstly deleted. The papers unrelated to the anticancer properties of gallnuts were then excluded. Patents, abstracts, case reports, and abstracts in symposium and congress were excluded as they did not contain sufficient information for evaluation and comparison with other studies. The review articles did not contain the original data. The articles were reviewed to determined compatibility with the inclusion criteria above. Based on the above criteria, 14 articles were eligible to be evaluated (Figure 2). Characteristics of the studies have been summarized in Table 2.

## 4. Bioactive Metabolites from Gallnuts

The primary and secondary metabolic processes of natural products can produce huge numbers of chemical constituents [38]. Specifically, the genesis of gallnuts also consists of an additional interaction between the plant host and invasion parasites. The complex processes of gallnuts formation thus should generate abundance of bioactive metabolites, which includes compounds originating from plants, insects, and mutual stimulation.

**Figure 3:**
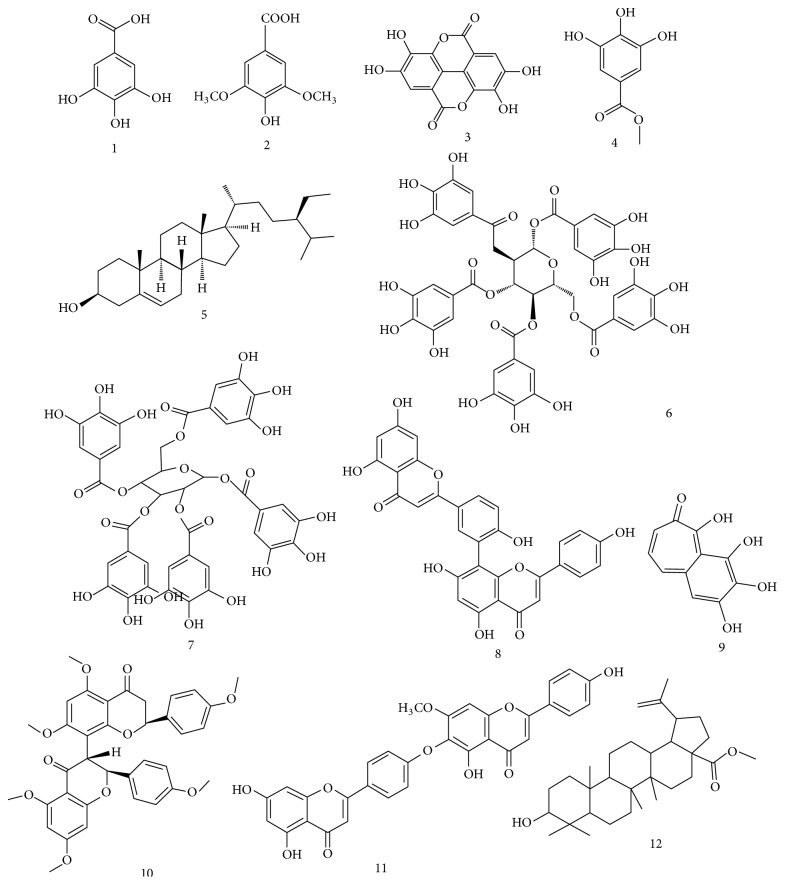
Chemical compounds identified from gallnuts.

Both Galla Chinensis and Galla Turcica contain a large amount (50–70%) of tannin (gallotannin), mainly of penta-undeca-galloylglucoses which have depside galloyl groups randomly distributed at the* C*-2,* C*-3, and* C*-4 positions on a penta-*O*-galloyl-*β*-D-glucose core [31]. According to the literature reported (Figure 3), twelve chemical components have been identified from gallnuts to the date, including gallic acid (1), syringic acid (2), ellagic acid (3), methyl gallate (4), *β*-sitosterol (5), 1,2,3,4,6-penta-*O*-galloyl-*β*-D-glucose (6), 1,2,3,4,6-tetra-*O*-galloyl-*β*-D-glucose (7), amentoflavone (8), purpurogallin (9), hexamethyl ether (10), isocryptomerin (11), and methyl betulate (12) [22, 32, 39–42]. Among them, gallic acid (1), ellagic acid (3), methyl gallate (4), *β*-sitosterol (5), 1,2,3,4,6-penta-*O*-galloyl-*β*-D-glucose (6), amentoflavone (8), and purpurogallin (9) have been reported to present anticancer activities in either* in vitro* or* in vivo* studies [22, 23, 34, 43–48]. However, the current researches of identification and isolation of gallnuts compounds were very few and mainly focused on the gallotannin only. The understanding of phytochemical knowledge for gallnuts thus clearly needs further complement.

The complex development of the plant-insect symbiont highlights the great potentials to produce a much higher variety of bioactive metabolites within gallnuts. In the future studies, the phytochemical researches are highly suggested to investigate the nontannin groups of gallnuts, such as flavones and terpenoids. Two flavones, amentoflavone and isocryptomerin, identified from Galla Turcica and the traditional anticancer uses of gallnuts indicated the possibility of abundance existence of flavones and other cytotoxic components.

## 5. Pharmacological Insights of Anticancer Aspect

Unlike many researches on the natural products, the anticancer effects of gallnuts were rarely studied using crude extracts. To the best of our knowledge, only Tong and his colleagues reported that the ether fraction of Galla Chinensis presented an* in vitro* inhibition ratio to the human glioblastoma cell U251 ranging from 42.34% to 72.54% in a dose-dependent manner, with IC_50_ being 12.76 *μ*g/ml. This activity was comparable to that of fluorouracil (from 40.6 to 75.5%, IC_50_ = 9.01 *μ*g/ml) [31]. Moreover, another anticancer-related study indicated that the ethyl acetate extracts of Galla Chinensis exhibited the significant inhibitory activities to the epidermal growth factor receptor, which was a validated target for different human malignancies, with IC_50_ values at 4.339 *μ*g/ml [33]. Both studies above somehow demonstrated that Galla Chinensis, as a medicinal agent individually, presented an effective anticancer activity in the laboratory tests.

As discussed previously, numbers of compounds identified from gallnuts have been reported to behave as anticancer agents in literature and thus could be considered as the material foundation of anticancer activities of gallnuts. Among them, gallic acid is the most reported compound shown to inhibit carcinogenesis in animal models and* in vitro* cancerous cell lines [24, 49, 50]. Ellagic acid is another polyphenolic compound that has been well researched for the anticancer properties. The growth inhibitory effects of ellagic acid have been determined on ranges of* in vitro* and* in vivo* experimental models of cancer [51–55]. Methyl gallate, isolated from three plants* Parthenocissus tricuspidata*,* Spondias pinnata,* and* Acacia hydaspica*, has also been reported to be cytotoxic to cancer cells* in vitro* [43, 56, 57]. Deiab et al. reported that 1,2,3,4,6-tetra-*O*-galloyl-*β*-D-glucose demonstrated significant antiproliferative effects on human MDA-MB-231 breast cancer cells with IC_50_ value as low as 1.2 *μ*M [34]. The bioflavonoid compound, amentoflavone, has been previously recognized as a cancer chemopreventive agent. It was found to stimulate the cell cycle arrest and apoptosis on many types of cancer cell lines* in vitro* [58, 59]. Taken together, the chemical profile somehow has conferred the anticancer property to gallnuts. Five compounds, including gallic acid, ellagic acid, methyl gallate, 1,2,3,4,6-tetra-*O*-galloyl-*β*-D-glucose, and amentoflavone, of gallnuts have been broadly reported to exhibit inhibitory effects against carcinogenesis through mechanisms of action on apoptosis, cell cycle, angiogenesis, or metastasis.

Gallic acid exerts anticancer activities through action on cell cycle, apoptosis, angiogenesis, invasion, and metastasis [48, 60, 61]. The molecular mechanisms of gallic acid identified in various types of human or rodent cancerous cells include modulation of apoptosis-related proteins, activation of ATM kinase, ribonucleotide reductase inhibition, cyclooxygenase inhibition, GSH depletion, UDP-glucose dehydrogenase inhibition, vascular endothelial growth factor (VEGF) inhibition, ADAMs inhibition, and NF-кB inhibition [25, 26, 30, 47, 62–67].

The ellagic acid is able to trigger the apoptosis and inhibit the proliferation, angiogenesis, migration, and invasion of cancer cells via regulation of several subcellular signaling pathways such as mitochondria-dependent signaling pathway, cell cycle signaling cascade, the protein kinase C signaling pathway, TGF-*β*/Smad3 pathway, and Wnt/*β*-catenin signaling pathway [46, 52, 53, 68–71].

As the results of methyl gallate treatment, the alteration of apoptotic molecules, and blockage of AKT, NF-кB, ERK1/2, PI3K, and JAK/STAT signaling pathways led to the apoptosis of cancer cells [43, 57]. In addition, methyl gallate also could exert antitumour activities through reversing immune suppression by inhibiting tumour infiltration of CD4^+^CD25^+^ regulatory T cells [72].

This impressive effect of 1,2,3,4,6-tetra-*O*-galloyl-*β*-D-glucose could be achieved through targeting on the overexpression of lactic acid dehydrogenase-A and metabolism genes of MDA-MB-231 cancer cells [34, 58]. The molecular mechanisms underlying the activities of amentoflavone mainly involve the action on human peroxisome proliferator-activated receptor gamma and mitochondria-emanated intrinsic pathways [73, 74]. Moreover, amentoflavone also could suppress the expression of NF-кB and VEGF and thus obstruct the tumour angiogenesis and metastasis [44, 75].

Arguably, these findings above partly provide the scientific evidence to support its traditional records of anticancer uses based on the TCM theory. However, the pharmacological research for anticancer properties of gallnuts is just at the beginning. The anticancer effects of individual gallnuts medicine need to be evaluated as a whole in a broad of* in vitro* and* in vivo* cancerous models. Their underlying mechanisms are expected to be elucidated in detail as well. More important, anticancer activity-guided isolation should be employed to systemically screen chemicals within gallnuts, aiming to obtain powerful anticancer agents or lead compounds.

## 6. Safety Verification

For the safety and healthy consideration, the use of gallnuts either orally or topically is not recommended over a long period at high doses [5]. However, both Galla Chinensis and Galla Turcica did not present significant, either acute or chronic, toxicity in the safety evaluative tests [32, 35].

In the toxicity studies using specific pathogen-free (SPF) Sprague-Dawley (SD) rats, the acute toxic test showed that the oral single dose of 5760 mg/kg of Galla Chinensis solution did not cause acute toxicity and the LD_50_ for rats was thus determined in excess of 5000 mg/kg. The subchronic toxicity study indicated that the no-observed effect of Galla Chinensis solution was lesser than 1500 mg/kg bw day, which is three times lower than that of the recommended dose of clinical uses. Moreover, Galla Chinensis solution did not produce any side effects on rats in central nervous system, cardiovascular system, and respiratory system [35].

In the acute toxicity test, the enema administration of Galla Turcica aqueous extracts at 10 g/kg did not cause any death or even significant adverse effects noted in the imprinting control region (ICR) mice. In the chronic use of Galla Turcica (up to 2 g/kg/day for 180 days), there are also no noticed changes in mice body weight and nutrient consumption as well as minor histological changes of organs such as the brain, heart, kidneys, lung stomach, liver, spleen, and uterus [32].

Though no significant toxicity in animal tests was reported, the gallnuts contain large amounts (over 50%) of tannins, which may cause negative health effects. Overdose intake of gall tannins active components could lead to the irritation of the gastric mucosa, nausea, vomiting, and even the fatal liver damage. Moreover, tannins were known as the metal ion chelators and digestive enzymes inhibitors. Therefore, they can cause anemia and dyspepsia in cases of long-term usage [5].

Overall, the gallnuts including Galla Chinensis and Galla Turcica could be considered safe in lower doses over a short period. However, the higher doses and longer term administration are not recommended.

## 7. Concluding Remarks

As the very special insect-plant symbiont in natural products, the gallnuts (Galla Chinensis and Galla Turcica) have been used as traditional herbal medicine for centuries. According to the TCM theory, gallnuts are claimed as the effective anticancer agents recorded in many ancient TCM encyclopedias and used in the clinical formulae in China. Based on the data accumulated in this review, it is primarily evident that gallnuts contain a number of bioactive phytochemical components, which at least partly account for the anticancer uses, and their medicinal uses are considered safe* in vivo*. The gallnuts thus could be proposed as a novel resource to develop the effective new chemotherapy drugs in future.

However, the number of current studies carried out on chemical constituents and pharmacological activities regarding the anticancer properties of gallnuts is surprisingly low. The following gaps are urgently noteworthy. First, as the complex symbiont formed through interaction between different living races, the current bioactive compounds identified from gallnuts should be just a corner of the whole chemical profile. Further study is required to systematically screen the chemical constitutes of gallnuts using bioassay-guided isolation to obtain more powerful anticancer components or lead compounds. Second, the pharmacological aspects of anticancer activities are barely investigated on either Galla Chinensis or Galla Turcica. Though anticancer actions of several compounds of gallnuts could be confirmed using information extracted from the research on other natural products, the pharmacological studies are still insufficient to determine their effects and to validate the ethno-anticancer uses. Both gallnuts themselves and pure bioactive compounds directly isolated from gallnuts thus should be comprehensively investigated into the molecule mechanisms underlying cancer effects to better understand the traditional medicine theory on gallnuts. Thirdly, anticancer activities of the extracts and compounds were mainly conducted to test in either* in vitro* or* in vivo* models, but not in clinical trials. Therefore, there are few reported data focused on clinical efficiency and side effects, and the results obtained may not be accurate and applicable in humans. Comprehensive well-controlled and double-blind clinical trials are therefore urgently required to reevaluate the efficacy and safety of anticancer uses of gallnuts.

## 8. Summary

Overall, the phytochemical and pharmacological studies reviewed herein are evident that gallnuts contain a number of anticancer phytochemical components and their medicinal uses are considered safe* in vivo*. It thus strongly underpins a fundamental understanding of anticancer activities of gallnuts medicine and supports their ongoing clinical uses in China. The further phytochemical evaluation, pharmacological investigation, and clinical trials are expected to progress gallnuts-based development to finally transform the wild traditional medicinal resource gallnuts to the valuable authorized anticancer drugs.

## Figures and Tables

**Table 1:** Anticancer uses in Chinese classical pharmaceutical books.

Gallnuts	Book	Medical uses	Dynasty	Year of publication	References
Galla Turcica	Tang Materia Medica	Diarrhea	Tang	659 AD	[9]

Galla Chinensis	Chinese Materia Medica Gleanings	Intestinal dysfunction, diarrhea	Tang	741 AD	[10]

Galla Turcica	Kai Bao Materia Medica	Ulcer, chancre, night sweats	Song	974 AD	[11]

Galla Turcica	Drug Properties	Uroclepsia	Tang	-	[12]

Galla Turcica	Oversea Materia Medica	Diarrhea, toxins	Tang	-	[13]

Galla Chinensis	Ri Hua Zi Materia Medica	Toxins	Song	-	[14]

Galla Chinensis	Kai Bao Ben Cao	Ulcer, haemorrhoids, chancre	Song	974 AD	[11]

Galla Chinensis	Commentaries on the Illustrations	Body fluid deficiency	Song	1061 AD	[15]

Galla Chinensis	Amplification on Materia Medica	Aphtha	Song	1116 AD	[16]

Galla Chinensis	Amplification on Materia Medica Addendum	Obstinate phlegm, heat	Yuan	-	[17]

Galla Chinensis	Materia Medica Meng Quan	Analgesic	Ming	1565	[18]

Galla Chinensis	Compendium of materia medica	Heat, toxins, phlegm, cough, wasting-thirst, night sweats, emesia, hemorrhage, analgesic, diarrhea, ulcer, chancre	Ming	1578	[19]

Galla Turcica	Fresh Chinese Materia Medica	Diarrhea, uroclepsia	Qing	1757	[20]

Galla Chinensis	Truth Chinese Materia Medica	Heat, phlegm, cough, diarrhea, haemorrhoids	Qing	1769	[21]

Galla Turcica	Truth Chinese Materia Medica	Heat, ulcer, diarrhea	Qing	1769	[21]

**Table 2:** Information summary of selected literature research for gallnuts.

Gallnuts	Active ingredients	Action related to anticancer	References
Galla Turcica	Purpurogallin	Inhibit the tyrosine-specific protein kinase of the human oncogene product epidermal growth factor receptor	[22]

Galla Turcica	50% ethanol extract	Protect against oxidative stress-induced liver injury	[23]

Galla Chinensis	Gallic acid	Increase the activities of phase II xenobiotic-metabolizing enzyme and decrease the activities of phase I xenobiotic-metabolizing enzyme, and thus decrease tumor incidence	[24]

Galla Chinensis	Gallic acid	Increase p53 and NF-*κ*B, decrease I-*κ*B protein, and thus induce apoptosis and inhibit cell viability in U937 cells	[25]

Galla Chinensis	Gallic acid	Upregulate p27(Kip1) level via disruption of p27(Kip1)/skp2 complex and the consequent degradation of p27(Kip1) and lead to G_2_/M phase arrest of MCF-7 cells	[26]

Galla Chinensis	1,2,3,4,6-Penta-*O*-galloyl-*β*-D-glucose	Regulate the expression of genes involved in cancer pyruvate metabolism, glycolysis/gluconeogenesis, and tyrosine metabolism	[27]

Galla Chinensis	Gallic acid	Downregulate expression of COX-2 and stathmin and inhibit the proliferation of 5-8F cells	[28]

Galla Chinensis	1,2,3,4,6-Penta-*O*-galloyl-*β*-D-glucose	Enhance cytotoxicity and PARP cleavage in cisplatin-treated Caki-2 cells	[29]

Galla Chinensis	Gallic acid	Suppress the matrix metalloproteinase-2/-9, protein kinase B and C signaling, and thus inhibit migration and invasion of U-2 OS cells	[30]

Galla Chinensis	Ether fraction	Inhibit the proliferation of U251	[31]

Galla Turcica	Aqueous extract	Show similar acute, chronic toxicity, and mortality to that of control	[32]

Galla Chinensis	Ethyl acetate extract	Inhibit the activities of epidermal growth factor receptor	[33]

Galla Chinensis	1,2,3,4,6-Penta-*O*-galloyl-*β*-D-glucose	Inhibit human lactic acid dehydrogenase-A and attenuate proliferation of MDA-MB-231 cells	[34]

Galla Chinensis	Water extract	Do not produce significant acute and chronic toxicity until overdose (3 times higher than that of the recommended dose), no side effects to rats in central nervous system, cardiovascular system, and respiratory system	[35]
